# Dual-Level Augmentation Radiomics Analysis for Multisequence MRI Meningioma Grading

**DOI:** 10.3390/cancers15225459

**Published:** 2023-11-17

**Authors:** Zongyou Cai, Lun M. Wong, Ye Heng Wong, Hok-lam Lee, Kam-yau Li, Tiffany Y. So

**Affiliations:** Department of Imaging and Interventional Radiology, The Chinese University of Hong Kong, Hong Kong SAR, China; caizongyou@link.cuhk.edu.hk (Z.C.); lun.m.wong@cuhk.edu.hk (L.M.W.); yehengwong@cuhk.edu.hk (Y.H.W.); isaacleeh16@gmail.com (H.-l.L.); 1155192547@link.cuhk.edu.hk (K.-y.L.)

**Keywords:** radiomics, meningioma, magnetic resonance imaging, data augmentation, machine learning

## Abstract

**Simple Summary:**

Prediction of high-grade meningioma on preoperative Magnetic Resonance Imaging (MRI) is essential in therapeutic planning and evaluation of prognosis. In this study, we propose a dual-level augmentation strategy incorporating image-level augmentation (IA) and feature-level augmentation (FA) to tackle class imbalance and improve the predictive performance of radiomics for meningioma grading on multisequence MRI. The radiomics model yields robust performance in 100 repetitions in 3-, 5-, and 10-fold cross-validation. In addition, our method significantly outperformed single-level augmentation (IA or FA) or no augmentation in each cross-validation. As an effective meningioma grading tool, our radiomics model may support clinical decision making and individualized treatment.

**Abstract:**

Background: Preoperative, noninvasive prediction of meningioma grade is important for therapeutic planning and decision making. In this study, we propose a dual-level augmentation strategy incorporating image-level augmentation (IA) and feature-level augmentation (FA) to tackle class imbalance and improve the predictive performance of radiomics for meningioma grading on Magnetic Resonance Imaging (MRI). Methods: This study recruited 160 consecutive patients with pathologically proven meningioma (129 low-grade (WHO grade I) tumors; 31 high-grade (WHO grade II and III) tumors) with preoperative multisequence MRI imaging. A dual-level augmentation strategy combining IA and FA was applied and evaluated in 100 repetitions in 3-, 5-, and 10-fold cross-validation. Results: The best area under the receiver operating characteristics curve of our method in 100 repetitions was ≥0.78 in all cross-validations. The corresponding cross-validation sensitivities (cross-validation specificity) were 0.72 (0.69), 0.76 (0.71), and 0.63 (0.82) in 3-, 5-, and 10-fold cross-validation, respectively. The proposed method achieved significantly better performance and distribution of results, outperforming single-level augmentation (IA or FA) or no augmentation in each cross-validation. Conclusions: The dual-level augmentation strategy using IA and FA significantly improves the performance of the radiomics model for meningioma grading on MRI, allowing better radiomics-based preoperative stratification and individualized treatment.

## 1. Introduction

Meningiomas are tumors that arise from the arachnoid cap cells, and they are the most common primary intracranial and central nervous system tumor [1]. Histopathological grading is a strong predictor of tumor progression, recurrence, and overall prognosis, and therefore it is crucial in therapeutic decision making and follow-up management [2]. Although most meningiomas are low-grade (WHO grade I) [3,4] and can be treated with surgery or, in some cases, radiotherapy without significant side effects [5], high-grade (WHO grade II and II) meningiomas often require a combination of the two therapies or more aggressive and careful treatment planning [5,6]. Magnetic Resonance Imaging (MRI) has been widely used to diagnose meningiomas, and features such as heterogeneous appearance, heterogeneous enhancement, perilesional edema, irregular margins, intratumoral hemorrhaging, bone destruction [2], and lower apparent diffusion coefficient (ADC) values [5,7] may suggest increased aggressiveness of the tumor. However, these features are not unique or reliable to differentiate between low-grade and high-grade tumors.

Radiomics can extract high-level characteristics from regions of interest (ROI) within a tumor and mathematically quantify these characteristics to aid in diagnosis, classification, or prognostication [8,9,10]. Therefore, radiomics may be beneficial to quantify important tumoral features, such as gray-level heterogeneity, shape, heterogeneity, intensity, position, and texture [5], in heterogeneous tumor analysis. Recent radiomics-based models have shown high potential capability in predicting meningioma grade [11,12], gene expression patterns [11,13], and prognosis [12,13]. However, the performance of radiomics models can be easily affected by class imbalance [14,15,16,17,18], and unequal representation of classes can bias model learning and performance, particularly when there is no dramatic difference between classes. Reflecting disease prevalence, clinical datasets of meningiomas are predominantly composed of low-grade data, with a much smaller percentage of high-grade cases. To deal with class imbalance, a common choice for radiomics is under- or oversampling [14,15,16], such as oversampling with the synthetic minority oversampling technique (SMOTE) [16], which synthesizes pseudosamples to balance the discrepancy of class. However, the performance of such sampling methods is limited by data variety, because they act at the feature level to introduce synthetic pseudosamples, which are along the direction of neighboring samples from the minority class. Synthetic pseudosamples can therefore lack sufficient representations of variety, making the minority class more general and difficult to distinguish from other classes. Image-level augmentation (IA) may be an appropriate solution to synthesize pseudosamples raised from a variety of potential perturbations at the image level, which cannot be reflected in feature-level augmentation (FA). IA is widely used in deep learning methods to avoid overfitting, while it has been rarely used in prior conventional radiomics research. In prior studies by Burak et al. [19] and Mitsuteru et al. [20], natural augmentation of data was performed by extracting features from different slices of the imaged volume. This method may only be performed in tasks where extracted features arise from separate imaging slices. However, we also recognize that shape-based features in radiomics contain both two-dimensional (2D) and three-dimensional (3D) information based on a volume mask. Michael et al. [21] and Sarv et al. [22] proposed a data augmentation for information transfer (DAFIT) approach, in which Gaussian noise was added on Computed Tomography (CT) and MRI, and the augmented datasets were incorporated in the prediction model. However, we consider that image augmentation methods may create other variations in MRI images besides variation in Gaussian noise. Makowski et al. [23] used different augmentation methods (e.g., deformation, contrast, brightness, and noise augmentation) in their study in prostate MRI, but these methods were based on natural image augmentation, rather than MRI-specific augmentation. No prior studies to date have investigated IA in conventional radiomics for brain tumors. The method of combining MRI-specific augmentation, such as elastic deformation, motion, and bias field augmentation for conventional radiomics in brain tumors, needs to be explored. Moreover, the imbalance ratio of classes remains after IA, and therefore, the incorporation of both IA and FA is essential.

In this study, we proposed a dual-level augmentation (IAFA) strategy to combine IA and FA to improve model performance. Furthermore, instead of a naïve train–test split, we used repeated cross-validation (CV) to evaluate the CV area under the receiver operating characteristic curve (CV-AUC) to better represent the capability of the model. In addition, we designed comparisons to demonstrate the advantage of the dual-level augmentation strategy compared with previously published methods. To our knowledge, this is the first radiomics study combining IA and FA to build more effective and robust models for brain tumor grading.

## 2. Materials and Methods

### 2.1. Data Acquirement

This study was approved by our institutional ethics committee with waiving of informed consent. This study was conducted in accordance with the Helsinki Declaration of 1975, as revised in 2013 [24]. We retrospectively recruited 193 consecutive patients with 193 pathologically proven meningiomas based on the following inclusion criteria: (1) patients aged 18 to 65 years, regardless of sex; (2) diagnosed and underwent surgical resection at our institution between 1 May 2007 and 1 May 2022. A total of 33 patients were excluded for the following criteria: (1) no MR within half year before surgical resection (n = 15); (2) recurrent meningioma (n = 4); (3) lacking operation report or tumor pathology report (n = 10); (4) previous surgery or biopsy before MRI (n = 1); (5) insufficient sequence images, such as without contrast-enhanced T1-weighted imaging (n = 3); (6) previous radiotherapy, chemotherapy, or chemoradiotherapy after diagnosis and before MRI (n = 0); (7) poor image quality, with images degraded by artefact (n = 0). Ultimately, 160 meningiomas (129 low-grade cases; 31 high-grade cases) were included in the study. Pathological grading was determined with assessment of histological and cytomorphical criteria according to the updated 2021 WHO classification system [25].

All MRIs were performed within half a year prior to surgery to limit mismatch between imaging findings and histopathology at resection. Unenhanced (T2-weighted, T2W) and contrast-enhanced (T1-weighted with contrast, T1C) sequences were included. All scanners used an eight-channel sensitivity-encoding head coil. The details of the multisequence image parameters are shown in Table 1.

### 2.2. Imaging Registration and Label Delineation

To avoid bias due to varying image acquisition parameters from different scanners, image registration was performed by registering T1C to T2W imaging using ITK-SNAP (V3.8.0) [26], given that T1C sequences were volumetric acquisitions and/or of smaller slice thickness and therefore contained more imaging information than T2W imaging. The registration was implemented by multiresolution schedule (coarsest level, 4; finest level, 2) based on a rigid transformation model with mutual information metrics and linear interpolation in ITK-SNAP [26]. Subsequently, the tumor core was manually delineated on each slice of the registered T1C images by an experienced neuroradiologist (T.Y.S., more than 10 years of work experience) to form the final form of segmentation as a base mask. For meningiomas, the tumor boundary can be best delineated solely on T1C in the overwhelming majority of cases, but T2W images were used for reference in all cases and for delineation of tumor boundaries with no enhancement on T1C images. For interobserver reproducibility analysis, three researchers (Y.H.W., H.L., and K.L.) collaborated to segment the tumor region, and another primary researcher (Z.C.) refined the segmentations. All tumor region delineation and refinement were performed using ITK-SNAP [26]. The mean (standard deviation) Dice Similarity Coefficient (DSC) between the base mask and the interobserver mask was 0.96 (0.02).

### 2.3. Image-Level Augmentation

The IA consisted of morphological operations and intensity operations. These operations consisted of MRI-specific augmentations, namely elastic deformation, motion and bias field augmentation, rotation and contrast, and noise augmentation [27,28,29], to simulate real-world imaging variations. Morphological operations empirically included 3 random affine rotations and 1 random elastic deformation, and intensity operations empirically included 1 random motion, 1 random bias field, 1 random noise, 1 random blur, and 1 random gamma augmentation. Morphological operations by character consist of augmentation with different rotations or deformations. Rotations and deformation of morphological operations not only affect the shape-based features but also all other categories of features; however, they do not cause changes in image intensity. Intensity operations similarly do not affect shape-based features. The public open-source image augmentation package torchio (V0.18.39) [30] was used to perform the 9 operations presented above on the preprocessed data.

### 2.4. Radiomics Features Extraction

The public open-source feature extraction package pyradiomics (V3.0.1) [31] was used to preprocess images and extract radiomics features from the base mask. Before extracting the features from the multisequence images, all images were normalized by resampling spacing to 1 × 1 × 1, *z*-score transformation, and intensity discretization with 32 bin width.

We applied 8 imaging filters, namely the original filter, wavelet filter, Laplacian of Gaussian (LoG) filter (sigma equals 1, 3, and 5), square filter, square root filter, logarithm filter, gradient filter, and exponential filter. For each imaging filter, texture features of 5 texture categories were further extracted, namely gray-level co-occurrence matrix (GLCM) features, gray-level run-length matrix (GLRLM) features, gray-level size-zone matrix (GLSZM) features, neighboring gray-tone difference matrix (NGTDM) features, and gray-level dependence matrix (GLDM) features. The extracted features included features of the following classes: 18 first-order features, 3 2D shape features, 11 3D shape features, and 75 texture features. There were 1595 features extracted from each input lesion on the T2W and T1C images, resulting in a total of 3190 features. The intraclass correlation (ICC) for all extracted radiomics features was 0.92. To avoid the influence of IA on feature reproducibility, we obtained the ICC for the radiomics features of each training set in cross-validation after IA and eliminated the poorly reproducible features (ICC less than 0.9) before performing FA.

### 2.5. Feature-Level Augmentation

The random oversampling technique SMOTE was applied to oversample the minority class (high-grade) from 234 samples to 918 samples to achieve a 1:1 ratio between the low- and high-grade data pool in the training set of CV. SMOTE was implemented using the public open-source image transformation package Imbalanced-learn (V0.10.1) [32]. We used five nearest neighbors. From the five nearest neighbors, only three neighbors were selected, and one sample was generated in each direction, since the required oversampling in the minority class is 300%. The synthetic samples were generated with the following steps: First, the feature difference between each minority sample and any three of its neighbors was obtained. Then, this difference was multiplied by a random number between 0 and 1 and added to the features of the corresponding samples. This resulted in the selection of random points along the line segment between each minority sample and any three of its neighbors. As a result of IA, there were more choices for the neighbors of the minority samples, therefore allowing the synthesized samples of the minority class to become more distinguishable compared with implementing SMOTE without prior IA.

### 2.6. Feature Selection Methods

From prior meningioma grading studies, it is known that multilevel feature selection can give better results. Fifteen feature selection methods were selected based on previous related research [13,31,33,34,35,36], including the filter methods Chi-square (CHSQ), *t*-test (TSQ), Kruskal–Wallis H-test tests (KWH), variance (VAR), relief (RELF), mutual information (MI), minimum redundancy maximum relevance ensemble (mRMRe) and the embedded methods L1-based logistic regression (L1-LR), elastic net (EN), least absolute shrinkage and selection operator (LASSO), L1-based linear support vector machine (L1-SVM), random forest (RF), extra tree ensemble (ETE), gradient boosting decision tree (GBDT), and xgboost (XGB). In this study, the filter methods were used as the first level of screening to reduce the number of features, and the embedded methods were used as the second level of screening to obtain the final features. This configuration complements the limitations of embedded methods, as an abundance of redundant features could impact the selection performance of embedded methods.

### 2.7. Classification Methods

We evaluated the performance of 13 machine learning classifiers, including Gaussian Naïve Bayes (GNB), Multinomial Naïve Bayes (MNB), Bernoulli Naïve Bayes (BNB), K-Nearest Neighbor (KNN), random forest (RF), Bagging (BAG), decision tree (DT), gradient boosting decision tree (GBDT), Adaptive Boosting (Adaboost), xgboost (XGB), Linear Discriminant Analysis (LDA), logistic regression (LR), and support vector machine (SVM). All feature selection and classification methods were implemented using the scikit-learn (V0.23.2) [37], xgboost (V1.6.2) [38], skrebate (V0.62) [39], and mrmr (V0.2.6) [40] packages in Python (V3.7) [41].

**Figure 1:**
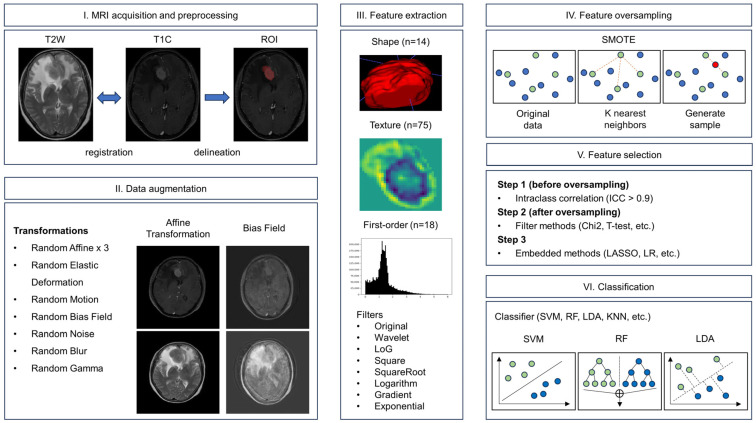
Flowchart illustrating the radiomics prediction pipeline.

To investigate the robustness of our proposed method based on different data allocation ratios, we used 3-, 5-, and 10-fold CV. We compared the performance of combinations of feature selection and classification methods in turn, which resulted in 728 combinations of feature selection and classification strategies. We obtained the optimal hyperparameters and the best combinations by repeating the experiments in 100 repetitions in 3-, 5-, and 10-fold CV, respectively. The details of the selected features of the best models in the 100 repetitions are shown in the Supplementary Materials (Tables S1–S3). The best combination of feature selection and classification methods was selected as the final model. A brief flowchart of the radiomics pipeline is given in Figure 1.

### 2.8. Comparison of Augmentation Methods

To understand the effects of augmentation on model performance, the four model settings, IAFA, IA, FA, and no augmentation (None), were compared using typical receiver operator characteristics (ROC) analysis. In each of the 100 repetitions, the performance of models trained in each of the 3-, 5- and 10-fold CV was computed as the area under the curve (AUC), as well as specificities that maximize the Youden index. These CV metrics obtained using the four aforesaid model settings were compared in terms of (1) performance, where the best-performing trial out of the 100 trials trained were compared, and (2) stability, where the distribution of performances across the 100 repetitions was compared, with smaller variance suggesting better stability. Experiments were completed with a 2.20 GHz Intel Core i7-8750H CPU with 16 GB memory.

The AUC from different train–test splits was calculated as

(1) $$AUC=f_{AUC}\left(Prob,Label\right)$$

where $f_{AUC}(∙)$ represents the AUC calculation function; Prob represents the probabilities of the test set in the naïve train–test split; and Label represents the labels of the test set in the naïve train–test split.

The mean AUC of k-fold CV, namely the mean AUC, which represents the conventional measure of k-fold CV AUC, was calculated as the average of AUCs across K folds:

(2) $$Mean\;AUC=\frac{1}{K}\sum_{k=1}^{K}f_{AUC}({Prob}_{k},{Label}_{k})$$

where $f_{AUC}(∙)$ represents the AUC calculation function; ${Prob}_{k}$ represents the probabilities of the test set in the fold; and ${Label}_{k}$ represents the labels of the test set in the fold.

The overall AUC, namely the CV-AUC, was evaluated by combining the model’s performance on all K testing folds in the same trial:

(3) $$CV-AUC=f_{AUC}\left(\left\{{Prob}_{k};k\in \left[1,K\right]\right\},\;\left\{{Label}_{k};k\in \left[1,K\right]\right\}\right)$$

The CV-Sensitivity and CV-Specificity were calculated based on the optimal point of the receiver operating characteristic curve (ROC) curve as follows:

*Sensitivity* = *TP*/(*TP* + *FN*) (4)

*Specificity* = *TN*/(*TN* + *FP*) (5)

where TP represents the true positive, the number of correctly predicted positive cases; FP represents the false positive, the number of incorrectly predicted positive cases; FN represents the false negative, the number of incorrectly predicted negative cases; and TN represents the true negative, the number of correctly predicted negative cases.

### 2.9. Statistical Analysis

Differences in age and gender between the tumor grade categories were compared using the Mann–Whitney U test and Fisher’s exact test, respectively. The best-paired CV-AUC were compared between settings using the two-sided DeLong’s test [42]. The distribution of paired CV-AUC, CV-Sensitivity, and CV-Specificity was compared between settings using the two-sided paired *t*-test. Tests of linear trends were performed for the paired CV metrics in each CV. Results were considered statistically significant when the *p*-value was less than 0.05. All analyses were performed using MedCalc (V20.211) [43].

## 3. Results

### 3.1. Clinical Characteristics of the Patients

A total of 160 patients were included, with a total of 129 WHO grade I meningiomas, 29 WHO grade II meningiomas, and 2 WHO grade III meningiomas. There were no significant differences in age or gender between patients with low-grade and high-grade tumors. The clinical characteristics of patients are summarized in Table 2.

### 3.2. Comparison of the Best Performance of the Four Paired Settings

The best models all consisted of CHSQ, LASSO, and LR in the different CVs. The best CV-AUC of our IAFA method in 100 repetitions was not less than 0.78 based on no more than 10 features in each CV. The corresponding CV-Sensitivities (CV-Specificity) were 0.72 (0.69), 0.76 (0.71), and 0.63 (0.82) in 3, 5, and 10-fold CV. The mean AUCs in 3, 5, and 10-fold CV were 0.75, 0.79, and 0.80, respectively. In addition, the mean (95% confidence interval, 95% CI) CV-AUCs of each CV in 100 repetitions were 0.71 (0.70–0.72), 0.73 (0.72–0.74), and 0.74 (0.74–0.75), respectively. The ranges of CV-AUC of each CV in 100 repetitions were 0.62–0.78, 0.66–0.79, and 0.68–0.78, respectively. The results of IAFA are summarized in Table 3.

**Figure 2:**
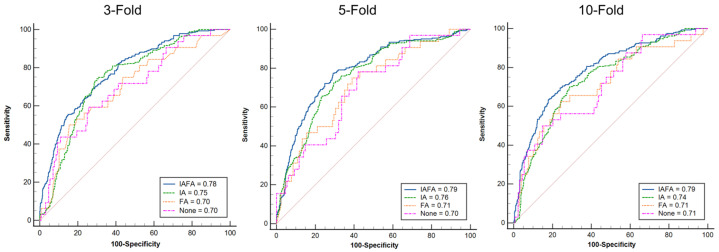
ROC curves of the four paired settings in different CVs: IAFA indicates the combination of the image-level augmentation and the feature-level augmentation. IA indicates image-level augmentation only. FA indicates feature-level augmentation only. None indicates no augmentation.

**Figure 3:**
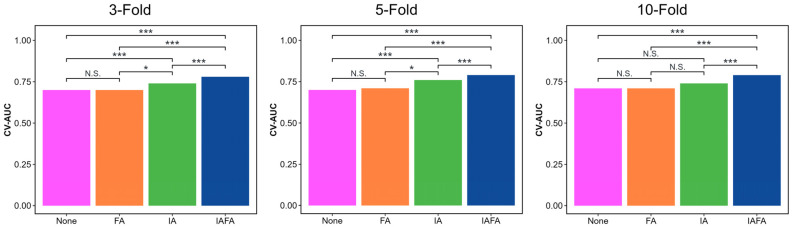
Bar charts of CVAUC of best-performing trials using different model settings: None indicates no augmentation; FA indicates feature-level augmentation; IA indicates image-level augmentation; IAFA indicates the combination of image-level augmentation and feature-level augmentation. * indicates a *p*-value less than 0.5; *** indicates a *p*-value less than 0.01; N.S. indicates not significant, i.e., *p*-value greater than or equal to 0.5.

Compared with other settings, the results of IAFA were significantly higher than other settings, while the results of FA setting were close to None. There was an increase in the best performance results from None to IAFA, as shown in Table 4. The ROC curves of the four paired settings in different CV folds are shown in Figure 2, and the corresponding CV-AUC was outputted in the legend of the ROC curve plots. The blue lines (IAFA) in each CV reach the color lines of the other settings in most various thresholds of the ROC curve, indicating the high performance of IAFA. Also, the Delong test [42] results of the four paired settings in the different CVs are reported in Figure 3. The results of IAFA were consistently statistically higher than other settings in each CV. In contrast, there were no significant differences between FA and None in each CV. There was no significant difference between IA and None or between IA and FA in the 10-fold CV.

### 3.3. Comparison of the Distribution of the Performance Results of the Four Paired Settings

**Figure 4:**
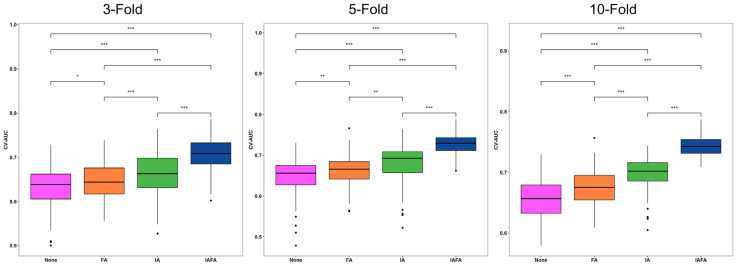
Distribution of CV-AUC results of the four settings from 100 repetitions in 3-, 5-, and 10-fold CV. * indicates a *p*-value less than 0.5; ** indicates a *p*-value less than 0.1; *** indicates a *p*-value less than 0.01. None indicates no augmentation; FA indicates feature-level augmentation; IA indicates image-level augmentation; IAFA indicates the combination of image-level augmentation and feature-level augmentation.

**Figure 5:**
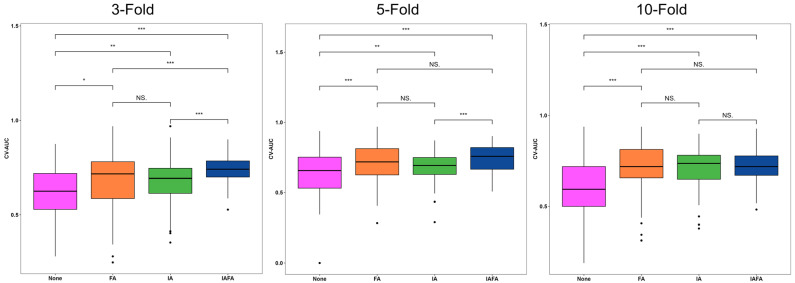
Distributions of CV-Sensitivity results of the four settings from 100 repetitions in 3-, 5-, and 10-fold CV. * indicates a *p*-value less than 0.5; ** indicates a *p*-value less than 0.1; *** indicates a *p*-value less than 0.01. N.S. indicates not significant, i.e., *p*-value greater than or equal to 0.5. None indicates no augmentation; FA indicates feature-level augmentation; IA indicates image-level augmentation; IAFA indicates the combination of image-level augmentation and feature-level augmentation.

**Figure 6:**
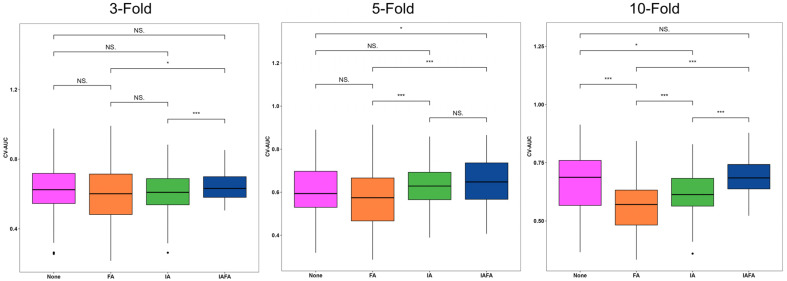
Distributions of CV-Specificity results of the four settings from 100 repetitions in 3-, 5-, and 10-fold CV. * indicates a *p*-value less than 0.5; *** indicates a *p*-value less than 0.01. None indicates no augmentation; FA indicates feature-level augmentation; IA indicates image-level augmentation; IAFA indicates the combination of image-level augmentation and feature-level augmentation.

The distributions of the CV metrics of the four settings from 100 repetitions in 3-, 5-, and 10-fold CV are shown in Table 5 and the boxplots (Figure 4, Figure 5 and Figure 6). The CV-AUC, CV-Sensitivity, and CV-Specificity of IAFA were consistently higher with lower standard deviation than the other settings in each CV (Table 5). There was a significant positive linear trend in the CV-AUC and CV-Sensitivity with a systematic increase in results from None, FA, IA to IAFA regardless of the number of folds.

## 4. Discussion

In this study, we proposed a dual-level IAFA strategy by combining IA and FA to tackle class imbalance and improve the performance of meningioma grade radiomics classification. Our method achieved no less than 0.78 CV-AUC in 3-, 5-, and 10-fold CV. Furthermore, in comparisons between IAFA, only IA, only FA, and no augmentation, IAFA significantly outperformed the other settings in each CV.

Previous literature has suggested radiomics to be promising to assist in meningioma grading, but reported performances (AUCs) widely range from 0.71 to 0.94 [11,12,13,33,44,45,46,47,48,49,50,51,52,53]. This may be due to the majority of studies utilizing only a naïve train–test split [11,12,13,33,44,45,46,47,49,51,52,53] validation, with only two exiting studies reporting the average result of cross-validation (CV) folds [48,50]. However, results derived from naïve train–test splits are susceptible to selection bias and variability, and may potentially overestimate the capability of the model. In this study, the AUC of the naïve train–test split showed quite a large range of performances, confirming that the results are influenced by data selection and different proportions of data allocation. The mean AUC, which represents the comparable measure of average cross-validation AUC with the literature, also increased with increasing CV folds, suggesting the metric to also be influenceable by data allocation. In contrast, the CV-AUC results in 100 repetitions were more stable across the different CVs. The CV-AUC of our method showed consistently high performance with a narrower range, suggesting CV-AUC to be a more stable and reliable estimate of model performance and capability. 

In paired comparisons of the best performances of the method, our results demonstrate that the dual-level IAFA strategy significantly improved the performance of the model. Additionally, the dual-level strategy showed consistent results across different CVs. IA helps the model overcome the challenge of data insufficiency but makes few contributions for class imbalance, while FA-synthesized features may lack robustness. A combination of IA and FA can simultaneously tackle these two challenges to balance the effect. The use of single IA or FA may not be able to independently optimize model performance as effectively. The CV-AUC of our method consistently outperformed the other settings in different CVs. In addition, the standard deviations of all metrics in our method across different CVs were consistently low compared with other settings, indicating an overall robust performance.

There were some limitations in this study. Firstly, our data were retrospective and derived from a single center. However, this may be a similar limitation to the majority of radiomics studies in meningioma grading to date. Nevertheless, our sample size, case composition within classes, and obtained results are comparable with those reported in the literature. Secondly, we only extracted features using tumoral ROIs, rather than peritumoral tissues, including peritumoral edema, which may be regions with potential significance for tumor grading and classification.

## 5. Conclusions

In this study, we proposed an effective and robust dual-level strategy to incorporate image-level augmentation and feature-level augmentation to mitigate class imbalance and map the performance landscape of radiomics for discriminating high- and low-grade meningiomas on MRI. The dual-level augmentation strategy improves both the performance and stability of the radiomics classification model.

## Figures and Tables

**Table 1:** Details of the sequence parameters obtained from multiple scanners.

Scanner	Philips Medical Systems						GE Medical System	
	Ingenia 1.5 T (n = 77)		Achieva 1.5 T (n = 9)		Achieva 3 T (n = 22)		SIGNA 3 T (n = 52)	
Parameters	T2W	T1C	T2W	T1C	T2W	T1C	T2W	T1C
Image Matrix	672 × 672	320 × 320 or 480 × 480	512 × 512	288 × 288	1024 × 1024	224 × 224 or 256 × 256 or 288 × 288	512 × 512	512 × 512
Slice no.	25–30	180–320	23–25	180	25–29	170–191	25–35	276–392
Spacing (mm)	(0.34, 0.34, 5.50)	(0.72, 0.72, 0.90) or (0.48, 0.48, 0.50)	(0.45, 0.45, 6.00)	(0.83, 0.83, 0.90)	(0.22, 0.22, 5.50	(0.89, 0.89, 0.90) or (0.80, 0.80, 0.90)	0.45, 0.45, 0.55	0.45, 0.45, 0.50
Slice Thickness (mm)	5	1–2	5	1.8	5	1.8	5	1
TR (ms)	5000–7000	25 or 33	4500–5000	25	2000–3100	25	3900–5100	6.10–6.20 or 11.70
TE (ms)	100	6–6.50 or 9.21	100	4.00–4.20	80	2.20–2.50	73–80	1.80–1.90
Acquisition Matrix	384 × 299 or 384 × 254 or 384 × 227)	256 × 256	372 × 279	268 × 268	512 × 390 or 420 × 335	224 × 222 or 256 × 256	460 × 460 or 416 × 416	256 × 256
Flip Angle (°)	90	30	90	30	90	30	142	12

**Table 2:** Patient demographics.

	Low-Grade	High-Grade		*p*-Value
	WHO Grade I	WHO Grade II	WHO Grade III	
Number (n)	129	29	2	-
Age (mean ± standard deviation, SD)	62.33 ± 10.35	64.00 ± 13.60	73.00 ± 6.36	0.11
Gender (n, %)				0.11
Male	43, 72.88	14, 23.73	2, 3.39	
Female	86, 85.15	15, 14.85	0, 0	
Brain invasion	0	13	1	-

**Table 3:** Performance of IAFA in 100 repetitions.

	The Proposed IAFA Method		
Data Size	160 Cases (129 Low Grade, 31 High Grade)		
Folds	3	5	10
Best trial in 100 repetitions			
Best combination	CHSQ, LASSO, and LR	CHSQ, LASSO, and LR	CHSQ, LASSO, and LR
Selected feature number	7–9	4–9	7–10
Mean AUC	0.75	0.79	0.80
Naïve train–test split AUC, range (train–test split)	0.68–0.88 (2:1)	0.66–0.94 (4:1)	0.62–0.99 (9:1)
CV-AUC	0.78	0.79	0.79
CV-Sensitivity	0.72	0.76	0.63
CV-Specificity	0.69	0.71	0.82
100 repetitions			
CV-AUC, mean (95% CI)	0.71 (0.70–0.72)	0.73 (0.72–0.74)	0.74 (0.74–0.75)
CV-AUC, range	0.62–0.78	0.66–0.79	0.68–0.79

95% CI indicates 95% confidence interval.

**Table 4:** Comparison of the best performance of the four paired settings from 100 repetitions in 3-, 5-, and 10-fold CV.

Best Paired CV-AUC				
Setting	None	FA	IA	IAFA
3-Fold	0.70	0.70	0.74	0.78
5-Fold	0.69	0.71	0.76	0.79
10-Fold	0.71	0.71	0.74	0.79

FA indicates feature-level augmentation; IA indicates image-level augmentation. IAFA indicates the combination of image-level augmentation and feature-level augmentation.

**Table 5:** Comparison of the mean and standard deviation of results from the four settings in 100 repetitions.

		Mean (Standard Deviation, SD)				*p*-Value
		None	FA	IA	IAFA	
3-Fold	CV-AUC	0.64 (0.04)	0.65 (0.04)	0.66 (0.05)	0.71 (0.03)	<0.01
	CV-Sensitivity	0.62 (0.14)	0.67 (0.15)	0.68 (0.11)	0.74 (0.08)	<0.01
	CV-Specificity	0.63 (0.14)	0.60 (0.16)	0.60 (0.12)	0.65 (0.09)	0.18
5-Fold	CV-AUC	0.65 (0.05)	0.66 (0.04)	0.68 (0.05)	0.73 (0.03)	<0.01
	CV-Sensitivity	0.64 (0.17)	0.72 (0.13)	0.69 (0.10)	0.75 (0.09)	<0.01
	CV-Specificity	0.61 (0.13)	0.57 (0.14)	0.63 (0.10)	0.65 (0.10)	0.23
10-Fold	CV-AUC	0.66 (0.04)	0.68 (0.03)	0.70 (0.03)	0.74 (0.02)	<0.01
	CV-Sensitivity	0.61 (0.14)	0.71 (0.13)	0.71 (0.10)	0.72 (0.09)	<0.01
	CV-Specificity	0.66 (0.13)	0.56 (0.11)	0.62 (0.10)	0.69 (0.08)	0.82

IAFA indicates combination of the image-level augmentation and the feature-level augmentation. IA indicates image-level augmentation only. FA indicates feature-level augmentation only. None indicates no augmentation.

## Data Availability

All codes generated from this project may be made available from the corresponding author upon reasonable request.

## Supplementary Materials

The following supporting information can be downloaded at https://www.mdpi.com/article/10.3390/cancers15225459/s1, Table S1: Features extracted from the best model in 100 repetitions in 3-fold CV; Table S2: Features extracted from the best model in 100 repetitions in 5-fold CV; Table S3: Features extracted from the best model in 100 repetitions in 10-fold CV.
