# Supplementary material for: Dual-Level Augmentation Radiomics Analysis for Multisequence MRI Meningioma Grading
Source: Cancers (Basel). 2023 Nov 17;15(22):5459. doi: 10.3390/cancers15225459 (PMC10670283; doi:10.3390/cancers15225459)
Supplement: Supplementary file 1 [file cancers-15-05459-s001.zip › cancers-2664883-supplementary.pdf]

Table S1. The features extracted from the best model in 100 repetitions in 3-Fold CV

3-Fold CV
**Fold 1**
original_firstorder_Kurtosis_t1
logarithm_glszm_GrayLevelNonUniformity_t2
wavelet-HHH_ngtdm_Coarseness_t2
log-sigma-5-0-mm-3D_firstorder_Uniformity_t2
wavelet-LLL_glszm_GrayLevelNonUniformity_t2
wavelet-LLL_glszm_ZoneEntropy_t2
wavelet-HHH_ngtdm_Strength_t2
log-sigma-5-0-mm-3D_glrlm_GrayLevelNonUniformityNormalized_t1
**Fold 2**
original_firstorder_Kurtosis_t1
log-sigma-5-0-mm-3D_glcm_ClusterProminence_t1
log-sigma-5-0-mm-3D_glszm_ZonePercentage_t2
logarithm_glszm_GrayLevelNonUniformity_t2
squareroot_glszm_GrayLevelNonUniformity_t2
log-sigma-5-0-mm-3D_glrlm_GrayLevelNonUniformityNormalized_t1
original_glszm_GrayLevelNonUniformity_t2
**Fold 3**
wavelet-LLL_firstorder_Kurtosis_t1
log-sigma-5-0-mm-3D_glrlm_GrayLevelNonUniformityNormalized_t1
log-sigma-5-0-mm-3D_glcm_MaximumProbability_t2
wavelet-HLL_firstorder_TotalEnergy_t1
wavelet-LLL_gldm_LowGrayLevelEmphasis_t2
wavelet-HLL_firstorder_Energy_t1
wavelet-HLH_firstorder_Energy_t1
wavelet-HLH_firstorder_TotalEnergy_t1
log-sigma-5-0-mm-3D_glcm_ClusterProminence_t1

Table S2. The features extracted from the best model in 100 repetitions in 5-Fold CV

5-Fold CV
**Fold 1**
log-sigma-5-0-mm-3D_glrlm_GrayLevelNonUniformityNormalized_t1
original_firstorder_Kurtosis_t1
log-sigma-5-0-mm-3D_glcm_MaximumProbability_t2
log-sigma-1-0-mm-3D_firstorder_TotalEnergy_t1
log-sigma-1-0-mm-3D_firstorder_Energy_t1
**Fold 2**
original_firstorder_Kurtosis_t1
log-sigma-5-0-mm-3D_glcm_ClusterProminence_t1
log-sigma-5-0-mm-3D_glrlm_GrayLevelNonUniformityNormalized_t1
squareroot_glszm_GrayLevelNonUniformity_t2

log-sigma-5-0-mm-3D_firstorder_Uniformity_t2
original_glszm_GrayLevelNonUniformity_t2
logarithm_glszm_GrayLevelNonUniformity_t2
**Fold 3**
original_firstorder_Kurtosis_t1
logarithm_glszm_GrayLevelNonUniformity_t2
log-sigma-5-0-mm-3D_glcm_SumSquares_t2
log-sigma-5-0-mm-3D_glrlm_GrayLevelNonUniformityNormalized_t1
wavelet-LLL_gldm_LowGrayLevelEmphasis_t2
original_glszm_GrayLevelNonUniformity_t2
log-sigma-5-0-mm-3D_glcm_ClusterProminence_t1
wavelet-LLL_glszm_SizeZoneNonUniformityNormalized_t2
squareroot_glszm_GrayLevelNonUniformity_t2
**Fold 4**
original_firstorder_Kurtosis_t1
log-sigma-5-0-mm-3D_glcm_ClusterProminence_t1
log-sigma-5-0-mm-3D_glrlm_GrayLevelNonUniformityNormalized_t1
log-sigma-5-0-mm-3D_firstorder_Uniformity_t2
**Fold 5**
original_firstorder_Kurtosis_t1
log-sigma-5-0-mm-3D_glcm_ClusterProminence_t1
logarithm_firstorder_InterquartileRange_t1
log-sigma-5-0-mm-3D_glrlm_GrayLevelNonUniformityNormalized_t1

Table S3. The features extracted from the best model in 100 repetitions in 10-Fold CV

10-Fold CV
**Fold 1**
original_firstorder_Kurtosis_t1
log-sigma-5-0-mm-3D_glrlm_GrayLevelNonUniformityNormalized_t1
log-sigma-5-0-mm-3D_glcm_ClusterProminence_t1
squareroot_glszm_GrayLevelNonUniformity_t2
log-sigma-5-0-mm-3D_firstorder_Uniformity_t2
logarithm_glszm_GrayLevelNonUniformity_t2
original_glszm_GrayLevelNonUniformity_t2
**Fold 2**
original_firstorder_Kurtosis_t1
log-sigma-5-0-mm-3D_glrlm_GrayLevelNonUniformityNormalized_t1
log-sigma-5-0-mm-3D_glcm_ClusterProminence_t1
log-sigma-5-0-mm-3D_glcm_MaximumProbability_t2
original_glszm_GrayLevelNonUniformity_t2
wavelet-LLL_glszm_ZoneEntropy_t2
squareroot_glszm_GrayLevelNonUniformity_t2
**Fold 3**
original_firstorder_Kurtosis_t1

log-sigma-5-0-mm-3D_glrlm_GrayLevelNonUniformityNormalized_t1
log-sigma-5-0-mm-3D_firstorder_Uniformity_t2
logarithm_glszm_GrayLevelNonUniformity_t2
wavelet-HLL_firstorder_Energy_t1
wavelet-HLL_firstorder_TotalEnergy_t1
original_glszm_GrayLevelNonUniformity_t2
log-sigma-5-0-mm-3D_glcm_ClusterProminence_t1
wavelet-LLL_gldm_LowGrayLevelEmphasis_t2
squareroot_glszm_GrayLevelNonUniformity_t2
**Fold 4**
original_firstorder_Kurtosis_t1
log-sigma-5-0-mm-3D_glrlm_GrayLevelNonUniformityNormalized_t1
logarithm_glszm_GrayLevelNonUniformity_t2
log-sigma-5-0-mm-3D_glcm_ClusterProminence_t1
log-sigma-5-0-mm-3D_firstorder_Uniformity_t2
wavelet-LLL_glszm_ZoneEntropy_t2
original_glszm_GrayLevelNonUniformity_t2
squareroot_glszm_GrayLevelNonUniformity_t2
**Fold 5**
original_firstorder_Kurtosis_t1
log-sigma-5-0-mm-3D_glrlm_GrayLevelNonUniformityNormalized_t1
original_glszm_GrayLevelNonUniformity_t2
log-sigma-5-0-mm-3D_firstorder_Uniformity_t2
log-sigma-5-0-mm-3D_gldm_GrayLevelVariance_t2
log-sigma-5-0-mm-3D_glcm_ClusterProminence_t1
log-sigma-5-0-mm-3D_glcm_MaximumProbability_t2
wavelet-LLL_glszm_SizeZoneNonUniformityNormalized_t2
squareroot_glszm_GrayLevelNonUniformity_t2
**Fold 6**
original_firstorder_Kurtosis_t1
log-sigma-5-0-mm-3D_glrlm_GrayLevelNonUniformityNormalized_t1
squareroot_glszm_GrayLevelNonUniformity_t2
log-sigma-5-0-mm-3D_glcm_MaximumProbability_t2
logarithm_glszm_GrayLevelNonUniformity_t2
log-sigma-5-0-mm-3D_glcm_ClusterProminence_t1
log-sigma-5-0-mm-3D_firstorder_Uniformity_t2
original_glszm_GrayLevelNonUniformity_t2
**Fold 7**
original_firstorder_Kurtosis_t1
log-sigma-5-0-mm-3D_glrlm_GrayLevelNonUniformityNormalized_t1
log-sigma-5-0-mm-3D_glcm_ClusterProminence_t1
log-sigma-5-0-mm-3D_firstorder_Uniformity_t2
logarithm_glszm_GrayLevelNonUniformity_t2
squareroot_glszm_GrayLevelNonUniformity_t2

original_glszm_GrayLevelNonUniformity_t2
**Fold 8**
original_firstorder_Kurtosis_t1
log-sigma-5-0-mm-3D_glrlm_GrayLevelNonUniformityNormalized_t1
log-sigma-5-0-mm-3D_glcm_ClusterProminence_t1
log-sigma-5-0-mm-3D_glcm_MaximumProbability_t2
logarithm_glszm_GrayLevelNonUniformity_t2
original_glszm_GrayLevelNonUniformity_t2
squareroot_glszm_GrayLevelNonUniformity_t2
**Fold 9**
original_firstorder_Kurtosis_t1
log-sigma-5-0-mm-3D_glrlm_GrayLevelNonUniformityNormalized_t1
log-sigma-5-0-mm-3D_glcm_ClusterProminence_t1
log-sigma-5-0-mm-3D_glcm_JointEnergy_t2
wavelet-HLL_firstorder_Energy_t1
wavelet-LLL_gldm_LowGrayLevelEmphasis_t2
wavelet-HLL_firstorder_TotalEnergy_t1
**Fold 10**
original_firstorder_Kurtosis_t1
log-sigma-5-0-mm-3D_glrlm_GrayLevelNonUniformityNormalized_t1
log-sigma-5-0-mm-3D_glcm_MaximumProbability_t2
squareroot_glszm_GrayLevelNonUniformity_t2
wavelet-LLL_glszm_ZoneEntropy_t2
logarithm_glszm_GrayLevelNonUniformity_t2
original_glszm_GrayLevelNonUniformity_t2
